# The Disability Cascade: A Preventable Consequence of the Loss of Disc Height following Lumbar Microdiscectomy

**DOI:** 10.7759/cureus.5169

**Published:** 2019-07-18

**Authors:** Adisa Kursumovic, Jeffrey M Muir, Joshua Ammerman, Richard Bostelmann

**Affiliations:** 1 Neurosurgery, Schwarzwald - Baar Klinikum, Villingen-Schwenningen, DEU; 2 Clinical Research, Telos Partners, Llc., Toronto, CAN; 3 Neurosurgery, Sibley Memorial Hospital, Washington, USA; 4 Neurosurgery, University Hospital Duesseldorf, Duesseldorf, DEU

**Keywords:** lumbar microdiscectomy, disc height, orthopaedics, lumbar spine, reoperation

## Abstract

Lumbar discectomy is a mainstay surgical treatment for herniation of the lumbar discs and is effective at treating radicular symptomology. Despite the overall success of the procedure; the potential for reherniation and reoperation is significant. To avoid this potential recurrence, surgeons often perform discectomy more aggressively, removing a larger volume of nuclear material in the hopes of minimizing the likelihood of reherniation. This approach, while beneficial in minimizing the chance of reherniation, is associated with a volumetric reduction of the nucleus within the disc space, making the disc more prone to collapse and thus inducing a significant post-operative loss of disc height. While potentially minor in isolation, the loss of disc height, in fact, impacts several aspects of overall patient well-being. We hypothesize that the loss of disc height following discectomy causes an increase in pain and subsequent disability, the combination of which ultimately impacts socioeconomic factors affecting both the patient and the healthcare system as a whole. In this report, we outline the evidence in support of this disability cascade and provide recommendations on methods for limiting its impact. Given the current focus on cost-effectiveness in healthcare decision-making, methods for limiting this potentially damaging sequence of events must be investigated.

## Introduction and background

Lumbar discectomy has been a cornerstone in the treatment of lumbar disc injury for the last four decades [1] and is a proven, effective procedure associated with generally positive results [2-4]. Fundamentally, removal of the herniated nuclear material is required to decompress the nerve and ease radicular symptoms, although the volume of material removed is left largely to the discretion of the surgeon. Conservative approaches to discectomy aim to remove a minimal amount of material, in the hopes of retaining as much nuclear material with the disc space as possible, to allow the disc to function normally, thus limiting the impact on the biomechanics of the vertebral motion segment. More aggressive approaches remove larger amounts of material, with a goal of minimizing the likelihood of reherniation. The key to successful discectomy, then, is to adequately balance these two aspects to maximize patient recovery.

Despite a record of success in addressing the symptomatology of lumbar disc herniation, significant complications such as reherniation have been reported in between 2% and 18% of cases [5-7], with higher rates of reherniation (27.3%) noted in studies of patients suffering from large defects [5]. Meta-analyses of randomized, controlled trials of aggressive versus traditional discectomy have demonstrated unequivocally that while aggressive discectomy is associated with significantly lower rates of reherniation, the rate of long-term recurrent back and leg pain is significantly higher [8-9]. Furthermore, the persistence of leg and/or back pain following surgery - the failed back surgery syndrome (FBSS) - is growing in incidence, with estimates of up to 2% observed in some studies [10]. FBSS is associated with post-operative quality of life declines greater than those seen in neuropathic pain disorders but also in other chronic diseases such as stroke or heart disease [11]. FBSS requires multidisciplinary care and represents a substantial barrier to long-term recovery [12]. As such, a trend in discectomy towards removal of larger volumes of disc material has emerged, in the hopes of minimizing the likelihood of reherniation and subsequent FBSS [6,13-14]. However, in a substantial proportion of patients - up to 36% by some estimates [14] - low back pain following this more aggressive form of discectomy persists.

While the removal of large volumes of material may decrease the likelihood of reherniation, it conversely increases the likelihood of loss of disc height and the risks associated with that loss and the accompanying alteration of the biomechanical competence of the vertebral motion segment, an observation that has been demonstrated both clinically [15-16] and in biomechanical laboratory settings [17-20]. The resulting consequence of this is a cascade of complications initiated by the loss of disc height, complications that can adversely affect the patient’s long-term health status. Here we discuss these complications and the need for a simple and reliable adjunct to discectomy that would allow for the minimum removal of disc material while providing the maximum protection against future reherniation.

## Review

The disability cascade

**Figure 1:**
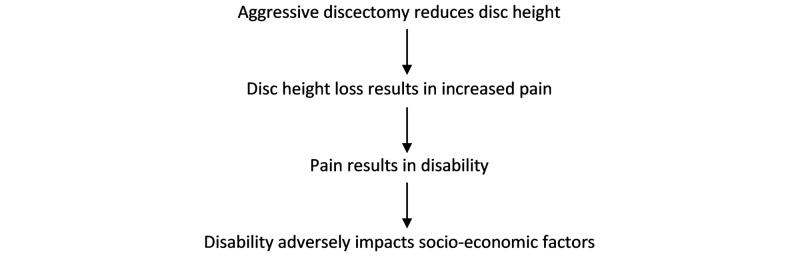
The disability cascade. The cascade is initiated by removal of significant volumes of nuclear material and ultimately ends with a substantial adverse effect on socioeconomic factors

While aggressive discectomy may be associated with lower rates of reherniation, we hypothesize that the removal of significant volumes of nuclear material during aggressive discectomy initiates a disability cascade that has substantial impacts on both patient-specific and system-wide factors of overall health. We hypothesize that aggressive discectomy removes excessive amounts of nuclear material and that this results directly in a loss of disc height post-operatively. Loss of disc height results in biomechanical deficits in the vertebral motion segment that support osteochondrosis and neuroforaminal stenosis, which subsequently impact disability, the ultimate consequence of which is an increase in the socioeconomic burden placed on both the patient and the healthcare system at large (Figure 1). 

Hypothesis evaluation

Discectomy and the Loss of Disc Height

Central to our hypothesis is the consistent observation that a consequence of aggressive discectomy is the post-operative loss of disc height [7,15-16,21]. In a study of 100 patients undergoing lumbar discectomy for refractory radiculopathy [7], McGirt et al. noted that patients who had greater volumes of disc material removed were associated with a greater degree of disc height loss at 12-month follow-up. Similarly, Lequin et al. [21] reported a correlation between the volume of disc material removed and the loss of disc height, even while the mean volume of nuclear material removed in this study was approximately 20% lower than that removed during other studies [7]. In prospective, comparison studies evaluating the effectiveness of discectomy versus other interventions or with discectomy combined with bone-anchored annular closure, the direct correlation between the volume of nuclear material removed and disc height loss has also been noted [16,22]. Elsewhere, a comprehensive study combining a clinical study and literature review of annuloplasty noted a direct correlation between the volume of disc material removed and disc height loss (p < 0.05) [23]. This study also noted that the loss of disc height continued the following discectomy, with significant decreases in disc height noted at 12-months post-procedure and continuing to decrease until 48-months post-discectomy. Also important in this study was the observation that disc height was not naturally recovered, with loss of height unchanged at 60-month follow-up.

Were the loss of disc height following discectomy an isolated finding, the trade-off between disc material removal and lowering rates of reherniation may be deemed worthwhile; however, there is compelling evidence to indicate that this decrease in post-discectomy disc height represents merely the first step in the disability cascade and triggers a sequence of events that ultimately adversely affect the patient’s long-term prognosis. 

The Relationship between Loss of Disc Height and Pain

The relationship between disc height and pain is multifactorial but studies have consistently observed that the loss of disc height has a significant impact on pain levels. This relationship is clearly demonstrated in studies of non-surgical spinal decompression, a form of motorized mechanical traction that reduces pressure within the intervertebral disc, thus limiting the extrusion of nuclear material through annular defects [24-25]. Studies of this technology have demonstrated a direct correlation between disc height and pain levels. Apfel et al. [26] examined disc height and pain levels in patients undergoing non-surgical spinal decompression for chronic low back pain and noted that, following a 6-week course of treatment, pain decreased by an average of 4.6 points on a visual analog scale rating pain from 1-10 (p < 0.001) and disc height increased from 7.5 mm to 8.8 mm (p < 0.001). They further noted that these outcomes were significantly correlated (r = 0.36, p = 0.04). The authors suggested that the improvement in pain associated with increased disc height may be mechanical in nature, with the decompressed discs reducing loads on both the disc and the adjacent facet joints. They also suggest that a cycle of pain and muscle spasm, involving surrounding musculature and the innervation patterns of the annulus and nucleus, may be responsible for the increases in pain that are noted with disc height loss, but they were unable to definitively explain their findings. This uncertainty underscores the difficulty associated with this concept; specifically, that while the relationship between pain and disc height is known, the causal mechanisms underlying this relationship remain to be fully elucidated. 

Several mechanisms have been suggested to be responsible for the increase in pain noted following the loss of disc height. Prominent among these mechanisms are changes in intradiscal pressure within the intervertebral disc itself that may be at least partially responsible for increased levels of pain. It is known that the integrity of the disc may influence, and be influenced by, the maintenance of hydrostatic pressures within the disc nucleus [17]. Degeneration of the disc causes decreased pressure within the disc, increasing the likelihood of overload and injury to the annulus. This relationship has been demonstrated in both cadaver and laboratory settings, with studies showing that disruption of annular integrity leads to significant alterations in post-discectomy pressure responses in both operative and proximal discs [17]. Indeed, the relative changes in intradiscal pressure have proven to be substantial, with operative discs seeing decreases in the pressure of up to 55%, while proximal discs have seen increases in the pressure of as much as 45% (p < 0.05). These dramatic changes in intradiscal pressure could be interpreted to indicate that conditions of high or low pressure lead to altered metabolic exchange that may also predispose operative and adjacent vertebral levels to disc pathology [19]. Finally, the relationship between disc height, radial disc bulge, and intradiscal pressure was examined in 15 human cadavers and it was found that disc height decreased and radial disc bulge increased in proportion to the mass of nuclear tissue removed during discectomy [18]. These authors also found that as the volume of nuclear material removed increased, there was a reciprocal decrease in intradiscal pressure. This inability to maintain intradiscal pressure was suggested as a causal factor in complications associated with loss of disc height, including pain, as the loss of height and intradiscal pressure limit the ability of the disc to maintain conditions required for normal physiologic function. 

Pain Results in Disability

A key consequence of increased pain - in spinal pathologies or pathologies in general - is the increased disability. This cause-effect relationship between pain and disability has been well illustrated in large, multinational studies such as the Global Burden of Disease 2010 Study [27-28], which examined the burden of low back pain and its connection to disability and quality of life. This comprehensive study ranked low back pain as the condition associated with the highest impact on disability, measured as years lived with disability (YLDs) and having the sixth highest impact in terms of disability-adjusted life years (DALYs). Pooling data from worldwide sources, the authors found that low back pain was ranked the greatest contributor to disability in 12 of 21 world regions and the greatest contributor to overall burden in 2 of the 21 world regions (western Europe and Australasia) [28].

Further evidence of the impact of pain on disability is seen in studies of lumbar discectomy. In a comprehensive study combining a systematic review of the literature and prospective data collection, Parker et al. [29] investigated the impact of post-discectomy pain on long-term patient-related outcomes and found, in both evaluations, that pain and disability were directly linked. Using Numeric Rating Scale Back Pain (NRS-BP) and ODI scores, the authors noted long-term pain (>24 months) in 34%-36% of patients in a robust pooled analysis of 90 studies and 21,180 patients [29]. The prospective portion of their study enrolled 115 patients that had a minimum 12-month follow-up after lumbar discectomy and assessed patient-related outcomes (PROs) at baseline and at 3-months, 1-year, and 2-years post-procedure. They noted that at both 1- and 2-year follow-up, 22% and 26% of patients, respectively, reported worsening of low back pain (NRS: 5.3 ± 2.5 versus 2.7 ± 2.8, p < 0.001) when compared with their 3-month results. Additionally, this cohort reported increases in disability (ODI%: 32 ± 18 versus 21 ± 18, p < 0.001) when compared with 3-month results, indicating the concurrent deterioration of both PROs. Similarly, McGirt et al. [7] likewise examined the effect of lumbar discectomy on pain, disability and quality of life in patients undergoing the first-time discectomy for refractory radiculopathy. At follow-ups of 6-weeks and 3, 6, 9, 12 and 24-months, the authors examined the impact of surgery on PROs including pain via visual analog scale (VAS) for both back pain (BP-VAS) and leg pain (LP-VAS), disability via ODI and quality of life via the SF-36 survey. They noted significant improvements in all measures at 6-weeks follow-up, with pain and disability (p>0.001 for all), improvements that were maintained through 24-month follow-up.

Increased Disability has a Socioeconomic Impact

The ultimate consequence of increased disability is the decreased productivity of the patient and the increased reliance on the healthcare system for treatment and support. These factors combine to illustrate the final step in the disability cascade, specifically the significant socioeconomic impact of patient disability. 

Regardless of diagnosis, increases in disability ultimately lead to greater requirements for care and lost productivity, be it at work or leisure productivity. Further analysis from the Global Burden of Diseases, Injuries and Risk Factors Study provides insight into this fact. In worldwide data collected for 359 diseases and injuries in 195 countries and territories over a 28-year period, overall increases in disability-adjusted life years (DALYs) for a number of conditions were observed, with non-communicable disease-associated DALYs increasing by 40% (38.6-43.0) between 1990 and 2017 [30]. These findings typify the issue of disability and its economic impact, as these additional years of life that may be gained by treatment are nevertheless lived in poorer health. The increased burden associated with greater numbers of patients living with disability thus has serious implications for both healthcare system planning and health-related expenditures [30]. 

Musculoskeletal disorders are among the leading causes of work disability and sickness absence from work, with some estimates suggesting that the total costs of this disability could be as high as $54 billion annually in the United States alone [31-32]. Other studies have estimated that up to 27% of retirements due to disability are due to musculoskeletal disorders [33]. Regarding discogenic lumbar disorders, the link is equally impactful and clear. The total costs (direct plus indirect) associated with non-surgical treatment of disc herniation have been calculated at $7,097, while mean costs per patient of $26,593 [34] to $34,242 [35] have been reported for surgical treatment of lumbar disc injury in several studies. The diagnosis and management of recurrent disc herniation thus have the potential to place a significant socioeconomic burden on both the patient and the healthcare system itself. This was illustrated directly by Klassen et al., [36] who reported that reoperation due to poor clinical outcomes of discectomy resulted in higher ODI scores and increased loss of work and length of hospital stay. This study compared patients undergoing lumbar discectomy with and without a bone-anchored annular closure device and noted significant differences in the rate of reoperation for reherniation (9% vs. 16%) and noted that, at 2-years post-procedure, patients who required reoperation registered ODI scores 2.9 times higher than those who did not require reoperation (46% vs. 16%), had VAS back scores 1.4 times higher (49% vs 35%) and VAS leg scores 3.6 times higher (25% vs. 7%). This increase in morbidity, characterized by increased levels of pain and disability, impacted both the direct and indirect costs associated with the episode of care, thus exerting a substantial economic impact. The total estimated direct medical costs associated with reoperation in this study was $952,348 ($13,802 per reoperated patient), of which control patients accounted for 59%. Indirect costs were also significantly higher among patients requiring reoperation, who missed 2.5 times more work and accounted for 37 times more in-patient hospital days. 

A final and important consideration when considering the impact of pain on the healthcare system is the effect of pain on the use of - and complications of - increased prescription medication use, specifically opioids. The Centers for Disease Control has identified opioid addiction as among the leading health crises in the United States today [37-38], and opioids remain among the most commonly prescribed analgesics for musculoskeletal pain [39-40]. The opioid crisis has worsened at an alarming rate in recent years, with opioid-related deaths increasing five-fold between 1999 and 2016 [37]. On average, 115 Americans die from an opioid overdose every day [37]. Any method for decreasing pain - especially pain syndromes that are associated with high opioid use - is therefore of inherent value. Post-discectomy treatment is not immune to this potential adverse event and caregivers must remain mindful of its effects when considering treatment for lumbar disc-related syndromes. Methods for improving lumbar discectomy and minimizing the loss of disc height and the initiation of the disability cascade thus have the potential to positively influence the current crisis, underscoring their importance. 

Summary

The balance between limiting reherniation risk and maintaining disc height presents the surgeon with significant challenges during lumbar discectomy. The potential for initiation of the disability cascade adds to these challenges and underscores the importance of identifying methods that allow for conservative removal of nuclear material while simultaneously limiting the likelihood of reherniation. To date, a definitive method for accomplishing this feat remains a challenge.

There is some evidence to suggest that materials that act as a nucleus pulposus analog may provide a method for limiting the cascade. One study [41] used an injectable nucleus hydrogel as a replacement for nuclear tissue lost to herniation following microdiscectomy. In this study, following standard microdiscectomy, the hydrogel material was injected into the nuclear void to replace the tissue lost during discectomy. The authors observed significant improvements in both leg and back pain following injection of the hydrogel, as well as improvements in functional ability. A significant drawback of this technology, however, was the postoperative release of the hydrogel, a result of the large annular defects that remained following surgery. While the early evidence for this technology is promising, and its ability to maintain disc height certainly suggests a role in limiting the initiation of the disability cascade, clinical studies remain in their infancy and more evidence is required to fully evaluate this treatment modality.

Alternative methods for minimizing recurrent herniation involve technologies designed to provide structural support to the disc following removal of the herniated material, thus limiting the likelihood of reherniation. These annular closure devices (ACDs) have been the subject of pre-clinical and early clinical studies [15,22,42], with the early evidence providing some evidence in support of their use. A recent meta-analysis performed [43] of four clinical studies [42, 44-46] investigating two current ACD technologies cited similar positive results from both devices, although the relatively limited clinical data prevented the authors from drawing firm conclusions on the overall effectiveness of these devices. Another recent randomized, controlled trial comparing lumbar discectomy plus bone-anchored annular closure with lumbar discectomy alone showed a significant improvement in all outcomes, including the rates of recurrent herniation (50% vs. 70%, p < 0.001), symptomatic reherniation (18% vs. 27%, p = 0.02) and reoperation (12% vs. 25%, p < 0.001) [47]. Similarly, positive results have been observed in real-world settings, such as a large, retrospective study of 171 patients who underwent discectomy with bone-anchored annular closure [48]. In that study, the rate of symptomatic reherniation in a cohort of patients with large annular defects was observed to be only 3.5% and all patients demonstrated clinically meaningful improvements in clinical outcome scores at both 3- and 12-months post-discectomy. Additionally, no reherniations were reported in the annular closure group, compared with a rate of 4.1% in a primary herniation group (p = 0.60). Finally, evidence indicates that ACDs may also play a role in maintaining disc height following discectomy, with one recent study reporting 90% disc height maintenance at 24-months post-procedure and 75% of pre-operative disc height maintained in fully 97% of patients at 12-months [22].

Despite these potentially positive results; however, there are reports of serious adverse events associated with the use of ACDs [49-50]. In one such case, the patient reported increased pain in the spine and extremities approximately 1 month following surgery [50]. Examination revealed bone resorption around the implant and signs of inflammatory changes in adjacent tissues. The patient underwent revision surgery, including removal of the implant and transpedicular and interbody fixation. In another documented case [49], a 32-year-old patient underwent annuloplasty using an ACD, which was stable for approximately 5 years postoperatively, at which point the patient presented with a new onset of low back pain radiating into the right leg. Imaging revealed the loosening of the implant, which was removed during revision surgery. These cases illustrate the limited long-term evidence regarding the use of ACDs. Additional data is required before definitive conclusions can be made on the long-term effectiveness of these implants, although the early evidence is encouraging.

## Conclusions

Regardless of the methods employed to halt or slow the cascade, the evidence indicates that the consequence of excessive removal of nuclear material during discectomy is the increase in pain and disability, which ultimately place a substantial socioeconomic burden on the patient and the healthcare system alike. Methods for minimizing the initiation of this cascade should be of the utmost importance to physicians. Research into preventative methodologies must continue.
